# The “no-touch” technique in the flexible ureteroscopic approach of renal stones

**DOI:** 10.25122/jml-2021-0217

**Published:** 2021

**Authors:** Bogdan Geavlete, Cosmin Cozma, Petrisor Geavlete

**Affiliations:** 1. Sanador Hospital, Bucharest, Romania; 2. Department of Urology, Sf. Ioan Emergency Clinical Hospital, Bucharest, Romania

**Keywords:** no-touch technique, ureteral access sheath, flexible ureteroscopy, postoperative complications, renal stone

## Abstract

Large meta-analyses demonstrated that ureteral access sheaths (UAS) have specific complications during and after flexible ureteroscopy (fURS). The present study focused on the technical aspects, advantages, drawbacks, and limitations of the latest “no-touch” technique (NTT) in the flexible ureteroscopic therapeutic approach of renal stones. A total of 288 patients with a single pyelocaliceal stone (largest diameter between 11 and 29 mm) underwent fURS: 144 using the 12/14 Fr UAS (group 1) and 144 without UAS (group 2). For NTT, we used four types of ureteroscopes: Olympus URF-V2 (8.5 Fr) – 33 cases, Storz Flex X2 (8.4 Fr) – 60 cases, single-use PUSEN PU 3022 (9.5 Fr) – 37 cases, and single-use PUSEN – PU 3033A (7.5 Fr) – 14 cases. For group 1, we used the Olympus URF–V2 ureteroscope in 44 cases, the Storz Flex X2 in 58 cases, and the single-use PUSEN PU 3022 in 42 cases. We compared the operative time, hospitalization periods, and complications. Successful access sheath insertion was noted in 83.3% of cases from group 1, and successful ureteroscope insertion was noted in 90.9% of cases from group 2. The average operative time was slightly higher in group 1 vs. group 2 (47 vs. 39 min). Stone-free rates (SFRs) were overall lower in group 2 (76.3% vs. 86.8%) at 1 month. At 3 months, we did not find a significant difference between these two groups. Superficial mucosal ureteral wall lesions were found in 38.8% of patients from group 1 and 4.1% from group 2. Hospitalization periods were longer in group 1 vs. group 2 (21 vs. 29 hours, respectively). The single-use 7.5 Fr ureteroscope should receive a special mention: the insertion was simple, we did not encounter any mucosal ureteral wall lesions, and all patients were discharged on the same day. Despite the clear advantages of routine UAS usage, there are many adverse events for the patient. Larger diameter sheaths involve a greater risk of ureteral wall injury. NTT seems to improve peri- and postoperative safety while preserving therapeutic efficiency. The new 7.5 Fr ureteroscopes appear to optimize surgical efficiency and diminish complications in the flexible ureteroscopic treatment of renal stones.

## Introduction

The prevalence of kidney stone’ disease elevated over the past three decades and reached a lifetime rate of approximately 14%. This increase was reported to affect most of the developed countries. Moreover, renal lithiasis appears to be related to multiple factors such as socioeconomic conditions, lifestyle changes, environmental features. It was also found to be strongly associated with specific comorbidities such as obesity, diabetes, and metabolic syndrome of high prevalence during the same time period [1].

With the rapid technological development of flexible ureteroscopy, supporting instruments were created while aiming to ease and facilitate this treatment modality, including the introduction of the ureteral access sheath (UAS) in 1974 as a method of passing a flexible ureteroscope into the ureter. Although UAS placement performance was poor during its initial introduction (19% of cases resulting in ureteral perforation), the use of this accessory instrument has now become an often-standard practice since the introduction of modern hydrophilic coated with hub-locking mechanisms UAS. With such modifications, the safety and wide use of UAS were well established, and these tools have commonly become part of the standard procedural steps of flexible ureteroscopy [2].

The introduction of a new generation of flexible digital ureteroscopes significantly improved the therapeutic and diagnostic efficacy of this endoscopic approach, allowing superior access to the upper urinary tract and facilitating wireless ureteroscopy. The present analysis was aimed to evaluate an alternative way of performing flexible ureteroscopy without the use of access sheaths or guidewires.

## Material and Methods

**Figure 1.**
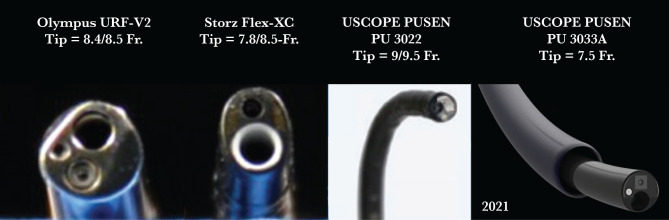
The flexible scopes used in our study.

A retrospective study was conducted over a period of 2 years (January 2019 – December 2020). A total of 288 consecutive patients with single pyelocaliceal stones (11–29 mm as the largest diameter) underwent fURS: 144 using the 12/14 Fr UAS (group 1) and 144 without UAS (NTT – group 2). For NTT, four types of ureteroscopes were used (Figure 1): Olympus URF-V2 (8.5 Fr) in 33 cases, Storz Flex X2 (8.4 Fr) in 60 cases, single-use PUSEN PU 3022 (9.5 Fr in 37 cases, and single-use PUSEN – PU 3033A (7.5 Fr) in 14 cases. In group 1, the Olympus URF-V2 ureteroscope was used in 44 cases, the Storz Flex X2 in 58 cases, and the single-use PUSEN PU 3022 in 42 cases.

In groups 1 and 2, the mean calculi diameter was 18 mm and 16 mm, respectively. Stones were localized mainly in the lower pole in both groups (87 in group 1 and 82 in group 2). The operative time, hospitalization periods, complications and success rates were compared between the two series.

**Figure 2.**
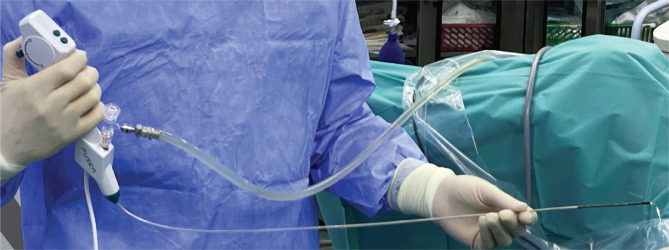
The new single-use digital flexible ureteroscope PU 3033A (7.5 Fr).

Concerning the technique used, in group 1, a guidewire was placed alongside the ureter, and the ureteral access sheath was placed fluoroscopically. The ureteroscope was subsequently introduced, followed by stone manipulation and lithotripsy. In group 2, the flexible ureteroscope was visually inserted and ascended, and the subsequent intrarenal disintegration of the calculi was carried out (Figure 2).

## Results

Successful access sheath insertion was achieved in 83.3% of cases in group 1, while the successful ureteroscope insertion was obtained in 90.9% of the patients in group 2.

In cases in which the access sheath or the ureteroscope could not be inserted, a double J stent was placed in order to recalibrate the ureter. The average operative time was slightly higher in group 1 compared to group 2 (47 versus 39 minutes, respectively), while the overall stone-free rate (SFR) at 1 month was lower in group 2 than group 1 (76.3% versus 86.8%, respectively). At 3 months, no significant difference was found between the two groups regarding the stone clearance rate. The stone-free status was not achieved in cases of lower pole calculi that were difficult to access. The average calculi diameter in which complete stone clearance was obtained was 16 mm in group 1 and 14 mm in group 2.

**Figure 3.**
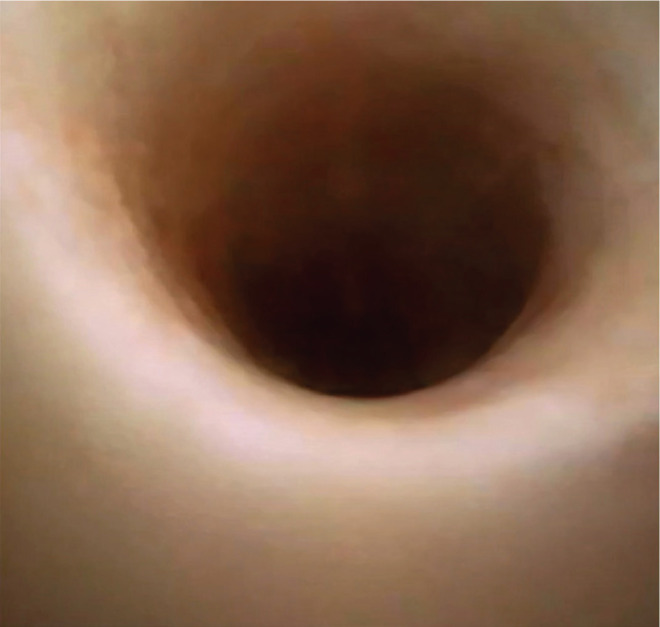
Normal aspect of the ureteral urothelium after using of the UScope PU3033A.

**Figure 4.**
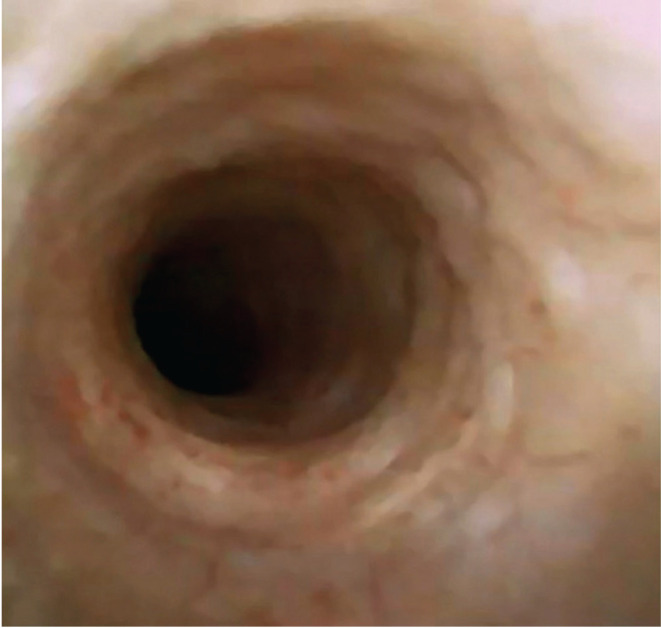
No lesions of the ureteral urothelium at the end of the ureteroscopic procedure.

The Clavien-Dindo system was used to rate the postoperative complications; we found 5 Clavien IIIA complications (4 in group 1 and 1 in group 2), 33 Clavien II (15 in group 1 and 18 in group 2), and 43 Clavien I (34 in group 1 and 9 in group 2) were encountered. Clavien IIIA complications were represented by renal colic requiring double J stenting, Clavien II adverse events consisted of sepsis (1 case), urinary tract infections (29 cases), and ureteral stent discomfort (3 cases), while Clavien I complications were hematuria and light ureteral stent discomfort. Superficial mucosal ureteral wall lesions were described in 38.8% of cases in group 1 and 4.1% cases in group 2 (Figures 3 and 4).

**Figure 5.**
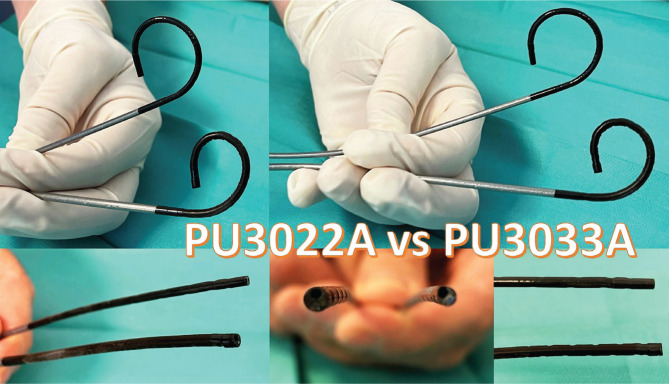
Single-use Uscope Pusen 7.5 Fr – PU3033A (the thinnest digital flexible ureteroscope in the world at this time) versus the single-use Pusen 9.4 Fr (PU3022A).

The average urinary tract infection rate was slightly higher in group 2 compared to group 1 (11.1% versus 9%, respectively). One case of sepsis was encountered in group 2 and was solved by intravenous antibiotics. The mean hospitalization period was longer in group 1 compared to group 2 (21 versus 29 hours, respectively). Regarding the single-use ureteroscope (7.5 Fr), the facile insertion, lack of mucosal ureteral wall lesions, and same-day discharge were outlined as main advantages (Figure 5).

## Discussion

The use of UAS during fURS allows accessing and evaluating the urinary collecting system in rapid repeated succession, at low intrarenal pressures, benefitting from superior visibility and improved drainage around the ureteroscope.

Despite these advantages of UAS placement, the current guidelines of the European Association of Urology have no clear recommendations for UAS usage during standard ureteroscopy procedures. In contrast, the American Urological Association guidelines recommend using UAS when performing retrograde intrarenal surgery for complex, high-volume renal stones. Despite the widespread acknowledgment of UAS as part of the common endourologic armamentarium, concerns and controversies remain surrounding the drawbacks of the routine use of these accessory instruments.

Based on the premises of continuous technological advances making the ureteroscopes thinner and more maneuverable, the technique of flexible ureteroscopy without the use of ureteral access sheath or safety guidewires was developed in recent years.

Concerning the stone-free rate, there is still a debate on whether UAS actually improves this parameter or not. In a multicenter prospective study, Traxer *et al.* reported that SFRs were overall lower consequent to the use of UAS (73.9% versus 82.8%). However, this difference was not statistically significant, and the authors concluded that UAS placement does not primarily constitute a method of increasing this particular surgical feature [3].

Following this perspective, Berquet *et al.* and Kourambas *et al.* emphasized no statistically significant difference in the SFR [4, 5]. On the contrary, L’esperance *et al.* showed in their retrospective study that UAS placement leads to significantly higher SFRs regardless of the calculi location in the pyelocaliceal system. However, subgroup analysis on stones per location in the renal pelvis and calyces showed no significance [6].

In a meta-analysis regarding the efficacy and safety of the ureteral access sheath during ureteroscopy, Huang *et al.* concluded that using a UAS during fURS has no significant effect on SFR, operative time, hospitalization time, or intraoperative complications. On the other hand, these accessory instruments significantly increase the incidence of postoperative complications. The study did not underline an obvious advantage of UAS using during ureteroscopy, thus concluding that this tool should not be routinely applied in all cases [7].

The present study showed a lower SFR at one month in the “no-touch” technique group, but no statistically significant difference was noted between the two series after three months.

The constant development of retrograde endoscopic treatment for renal calculi also involved widespread UAS use, while adverse events related to these adjunct tools became more frequent. Intraoperative complications associated with access sheaths usage include bleeding, perforation, and avulsion. Traxer and Thomas found superficial ureteral mucosal wall lesions in nearly half of the patients following the insertion of an access sheath, with 15% of the situations extending beyond the mucosa into the smooth muscle layer [7]. Additionally, the UAS use may also result in stricture formation and edema of the ureteral smooth muscle, eventually leading to ureteral obstruction.

Following this point of view, the present clinical analysis observed superficial ureteral mucosa lesions in 38.8% of the UAS patients and only in 4.1% of the “no-touch” technique cases. These findings demonstrated the fact that the access sheath is traumatic for the ureteral wall, an aspect that must be seriously taken into consideration while performing flexible ureteroscopy. Furthermore, single-use 7.5 Fr ureteroscopes provided the conditions for mucosal lesions to be completely avoided.

As far as intrarenal pressure is concerned, it was demonstrated that the use of UAS reduces the intrarenal pressure by facilitating the flow and irrigating out of the collecting system. *In vivo* studies on cadaveric human and porcine models emphasized that UAS placement is capable of reducing intrarenal pressure when compared to no UAS being applied at various irrigation pressures [8].

Also, the gradual reduction of the intrarenal pressure was shown to be associated with the increasing diameter of the access sheath. It was shown that a normal physiological intrarenal pressure could only be achieved when using the 14/16 Fr UAS. Following this line, some studies concluded that UAS placement during flexible ureteroscopy does decrease intrarenal pressures by comparison to not being mounted.

However, the degree of pressure reduction may not reach sufficient levels in order to prevent complications associated with high intrarenal pressures during surgery, especially under pressure irrigation. Furthermore, large-diameter UAS augments the frequency of associated complications. The newest ureteroscopes are thinner and enable a reduced intrarenal pressure to be maintained by draining the irrigant around the scope.

In any case, further studies are required to demonstrate the efficacy and safety of using a thinner ureteroscope without an access sheath from the point of view of intrarenal pressure. According to the presently obtained outcomes, it was underlined that a higher urinary tract infection rate was present among patients of the “no-touch” technique group, thus confirming the intrarenal pressure was higher in that particular setting.

As far as the double J stenting following ureteroscopy is concerned, it is advised that such a stent should be placed when ureteral access sheath is used in order to prevent obstruction, renal colic, deterioration of renal function, and postoperative complications. If ureteral wall injury occurs during ureteroscopy due to UAS placement, postoperative stenting was demonstrated to play a reparatory role in the prevention of ureteral edema while also minimizing pain secondary to residual stone fragments and blood clots [9]. When using a thin ureteroscope in the “no-touch” technical approach, the reasons for placing a postoperative stent are fewer, mainly due to the fact that there are smaller chances of producing a ureteral wall injury.

As part of the conventional technique, a safety guidewire is maintained alongside the ureteroscope during stone fragmenting in order to prevent from losing access [10]. On the other hand, Nakada *et al.* showed that wireless flexible ureteroscopy is safe, the only complications encountered being urinary tract infections (4 cases), urosepsis (2 cases), and urinary retention (one case) while no patient had ureteral wall injury or avulsion [11].

Dickstein *et al.* proved that no safety guidewire is actually necessary during uncomplicated ureteroscopy, with no intraoperative complications resulting from the lack of guidewire usage [12]. Moreover, Grasso *et al.* performed 227 procedures without using a safety guidewire and managed to successfully treat ureteral and renal stones as well as urothelial tumors [13].

It may be relevant to acknowledge that the new single-use PUSEN 3033A (7.5 Fr) ureteroscope was scarcely related to intra- or postoperative complications in the NTT series. Moreover, same-day discharge was noted in the case of all patients.

## Conclusion

Despite the clear advantages of UAS usage, there are many drawbacks in terms of safety and postoperative quality of life. Large diameter UAS involves a greater risk for ureteral wall injury. According to the available data, NTT seems to be remarkably efficient and safe. The new 7.5 Fr ureteroscope optimizes the success rate and diminishes complications during the ureteroscopic treatment of renal stones.

### Key-points

• recent studies show that using the ureteral access sheath has no effect on the stone-free rate;
• a significant percentage of patients suffered superficial mucosal ureteral wall lesions in the group in which UAS was routinely used;
• the newest ureteroscopes are thinner and enable a reduced intrarenal pressure to be maintained by draining the irrigant around the scope.

## Acknowledgments

### Conflict of interest

The authors declare that there is no conflict of interest.
